# An Augmented Lagrangian Based Compressed Sensing Reconstruction for Non-Cartesian Magnetic Resonance Imaging without Gridding and Regridding at Every Iteration

**DOI:** 10.1371/journal.pone.0107107

**Published:** 2014-09-12

**Authors:** Mehmet Akçakaya, Seunghoon Nam, Tamer A. Basha, Keigo Kawaji, Vahid Tarokh, Reza Nezafat

**Affiliations:** 1 Department of Medicine (Cardiovascular Division), Beth Israel Deaconess Medical Center and Harvard Medical School, Boston, Massachusetts, United States of America; 2 Surgical Technologies, Medtronic, Inc., Littleton, Massachusetts, United States of America; 3 Department of Medicine (Section of Cardiology), University of Chicago, Chicago, Illinois, United States of America; 4 School of Engineering & Applied Sciences, Harvard University, Cambridge, Massachusetts, United States of America; National Taiwan University, Taiwan

## Abstract

**Background:**

Non-Cartesian trajectories are used in a variety of fast imaging applications, due to the incoherent image domain artifacts they create when undersampled. While the gridding technique is commonly utilized for reconstruction, the incoherent artifacts may be further removed using compressed sensing (CS). CS reconstruction is typically done using conjugate-gradient (CG) type algorithms, which require gridding and regridding to be performed at every iteration. This leads to a large computational overhead that hinders its applicability.

**Methods:**

We sought to develop an alternative method for CS reconstruction that only requires two gridding and one regridding operation in total, irrespective of the number of iterations. This proposed technique is evaluated on phantom images and whole-heart coronary MRI acquired using 3D radial trajectories, and compared to conventional CS reconstruction using CG algorithms in terms of quantitative vessel sharpness, vessel length, computation time, and convergence rate.

**Results:**

Both CS reconstructions result in similar vessel length (*P* = 0.30) and vessel sharpness (*P* = 0.62). The per-iteration complexity of the proposed technique is approximately 3-fold lower than the conventional CS reconstruction (17.55 vs. 52.48 seconds in C++). Furthermore, for in-vivo datasets, the convergence rate of the proposed technique is faster (60±13 vs. 455±320 iterations) leading to a ∼23-fold reduction in reconstruction time.

**Conclusions:**

The proposed reconstruction provides images of similar quality to the conventional CS technique in terms of removing artifacts, but at a much lower computational complexity.

## Introduction

Non-Cartesian sampling trajectories in MRI such as radial [1] and spiral [2] imaging have a number of favorable properties compared to Cartesian sampling trajectory [3], which has lead to their use in a number of applications. For instance radial trajectories have been used for accelerated time-resolved MRI with constrained back projection reconstruction [4], [5], stack-of-radial and stack-of-spiral acquisitions have been utilized for 3D cardiac MR (CMR) [6], [7], and 3D radial acquisition with isotropic spatial resolution have been employed for scanning whole-heart CMR [8], [9], [10]. One of the main advantages of non-Cartesian trajectories is the incoherent artifacts generated as a result of undersampling [11], [12], [13]. Furthermore, the oversampling of the k-space center in radial and spiral trajectories provides superior performance with respect to motion of the object when compared to Cartesian sampling [14], [15]. The oversampling of the k-space center also provides a fully-sampled low resolution image, which can be utilized with parallel imaging techniques for accelerated acquisition [16], [17].

However, non-Cartesian trajectories require a more complicated reconstruction process compared to Cartesian trajectories. The gridding algorithm [18] is commonly used to reconstruct non-Cartesian data. While the reconstruction of Cartesian data requires inverse Fourier transform on the uniformly distributed samples in the rectilinear grid for each k-space dimension, gridding reconstruction performs convolution interpolation of the non-uniformly sampled data and re-samples them onto the rectilinear Cartesian grid in order to utilize the computationally efficient inverse Fourier transform. The density compensation of the non-uniformly distributed samples is also essential before the interpolation is performed [19],[20]. Although the gridding algorithm can efficiently reconstruct the data acquired with non-Cartesian trajectories, its performance deteriorates significantly for highly undersampled data [12].

There have been recent studies to apply compressed sensing (CS) technique to reconstruct undersampled MR data [12], [21], and it has been shown that CS efficiently removes incoherent undersampling artifacts. CS reconstructions for non-Cartesian trajectories have also been demonstrated with notable improvement over the conventional gridding reconstruction [11], [13], [22], [23], [24]. The CS reconstruction is typically performed using conjugate-gradient (CG) type iterative algorithms, for which the gridding and regridding operations are repeatedly performed during the iterative process [12]. However, the computational overhead of the iterative CS reconstruction for non-Cartesian trajectories results in prolonged reconstruction time. Parallel computing techniques using graphics processing units (GPUs) have recently gathered great interest in improving MRI reconstruction time [25], [26], [27]. GPU-accelerated implementations of CS reconstructions for non-Cartesian trajectories have been shown to substantially accelerate the reconstruction time by parallelized execution of the reconstruction process [28], [29]. For large MR data sets such as high resolution 3D whole-heart imaging, however, the amount of computation is still demanding to be clinically feasible even with the parallelized implementation [30], and therefore reducing the amount of computation in the reconstruction, especially in gridding and regridding operations, is highly desirable.

In this work, we sought to develop an alternative method for solving the CS reconstruction for non-Cartesian trajectories, which eliminates the need for gridding and regridding at every iteration, thereby reducing the computational complexity and the execution time of the CS reconstruction for non-Cartesian trajectories. Phantom and in vivo cardiac MRI examples are shown to demonstrate the feasibility of the proposed approach.

## Theory

Non-Cartesian data is typically reconstructed using a gridding algorithm [18], where first a trajectory-dependent density compensation function (DCF) is applied to each data point to compensate for the non-uniform sampling density [19], [20], [31]. Then the data points are convolved with a gridding kernel and re-sampled onto a Cartesian grid, which is inverse Fourier transformed to obtain an image. Finally, de-apodization is performed on this image via division by the apodization function, given by the Fourier transform of the gridding kernel function [18]. This procedure can be summarized as
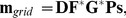
(1)where **m** *_grid_* is the reconstructed image, **s** is the measured non-Cartesian k-space data, **P** is a diagonal matrix representing the DCF, G^*^ is the gridding operator, F^*^ is the inverse fast Fourier transform (IFFT), and **D** is a the diagonal de-apodization operator.

The acquired non-Cartesian data can also be expressed in terms of an encoding matrix as
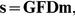
(2)where **m** is the image to be reconstructed, **G** is the regridding operator, **F** is the fast Fourier transform (FFT), and **D** is a the diagonal de-apodization operator as above. Unlike the conventional gridding algorithm, the density compensation is not required before the regridding because the density of the Cartesian grid is uniform [12], [32]. Without loss of generality, we ignore the de-apodization function, since it can be corrected for the final image estimate [3]. Iterative CS reconstruction solves a constrained minimization problem of the form
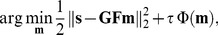
(3)where 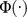 is a sparsity inducing constraint, typically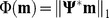, where **Ψ** is a sparsifying transform (e.g. image or wavelet domain) in which the image of interest is sparse, or 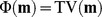, the total variation (TV) of the image. This is typically solved using conjugate-gradient type techniques [12].

### Proposed Algorithm

We take an alternative approach involving three steps: 1) We pose the problem in [3] as a constrained optimization problem using an auxiliary variable and minimize its augmented Lagrangian (AL) [33], 2) Rather than solving the AL directly, we use the less computationally expensive alternating directions method (ADM) [34], [35], 3) In the solution of one of the sub-problems of the ADM, we approximate the matrix G^*^G by a diagonal matrix.

We first introduce an auxiliary variable u, and equivalently write [3] as
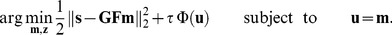
(4)The AL of [4] is given by 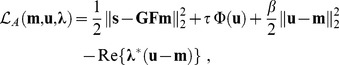
(5)where **λ** ^*^ is the conjugate transpose of the multiplier **λ**. At iteration *t*, the AL method performs the following updates
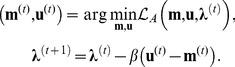
(6)The minimization in [6] is computationally challenging to perform jointly for **m** and **u**. However, it has been shown that in various CS applications, this could be performed with high accuracy using the more computationally efficient ADM [34], [35], [36], [37]. In this case, the ADM first fixes **m** and updates **u** (which corresponds to denoising with respect to the sparsity inducing constraint 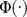), and then fixes **u** and updates **m** (which corresponds to data consistency). Thus the first step in iteration *t* of the proposed method becomes: 
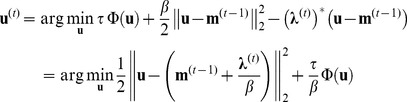
(7)for denoising. Note for 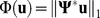 with a unitary transformation **Ψ** (e.g. image or wavelet domain), this step corresponds to *l*_1_ soft thresholding 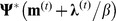 by *τ*/*β*, and transforming back to image domain by applying **Ψ**. This step can also be implemented for other regularizers such as TV [38] or more complicated techniques without closed-form expressions [39], [40]. The data consistency step is given by
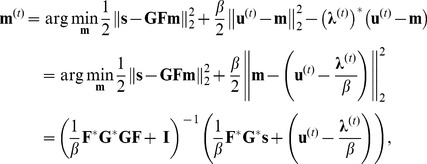
(8)which leads to a closed form expression, noting **I** is the identity matrix. As in [6], 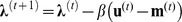 is the last step of the iteration.

The final innovation in the proposed technique is to evaluate [8] in a less computationally intensive way, by avoiding gridding and regridding operations, G^*^ and **G** respectively. First we note, the FFT of 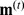 is easier to calculate and given by 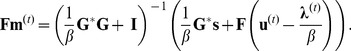
(9)The main savings of the proposed method in gridding and regridding operations come from avoiding the inversion of the first term. To do so, G^*^G is approximated by a diagonal operator. Noting that both gridding and regridding operators act locally, we hypothesize the contributions from off-diagonal elements will only be limited to a small number of data points. As such, we treat G^*^G as a diagonal matrix itself, and approximate 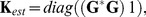
(10)where *diag*(·) assigns the elements of the vector in its argument to the diagonals of a diagonal matrix, and **1** is the all-ones vector. Estimation of G^*^G by **K** *_est_* allows us to avoid gridding and regridding at every iteration, and since gridding, G^*^ and regridding, **G** involve approximations themselves, the artifacts due to this diagonal estimation may not be very noticeable in the final reconstructed images. We note similar approximations have been used in the context of parallel imaging as well [41].

**Figure 1:**
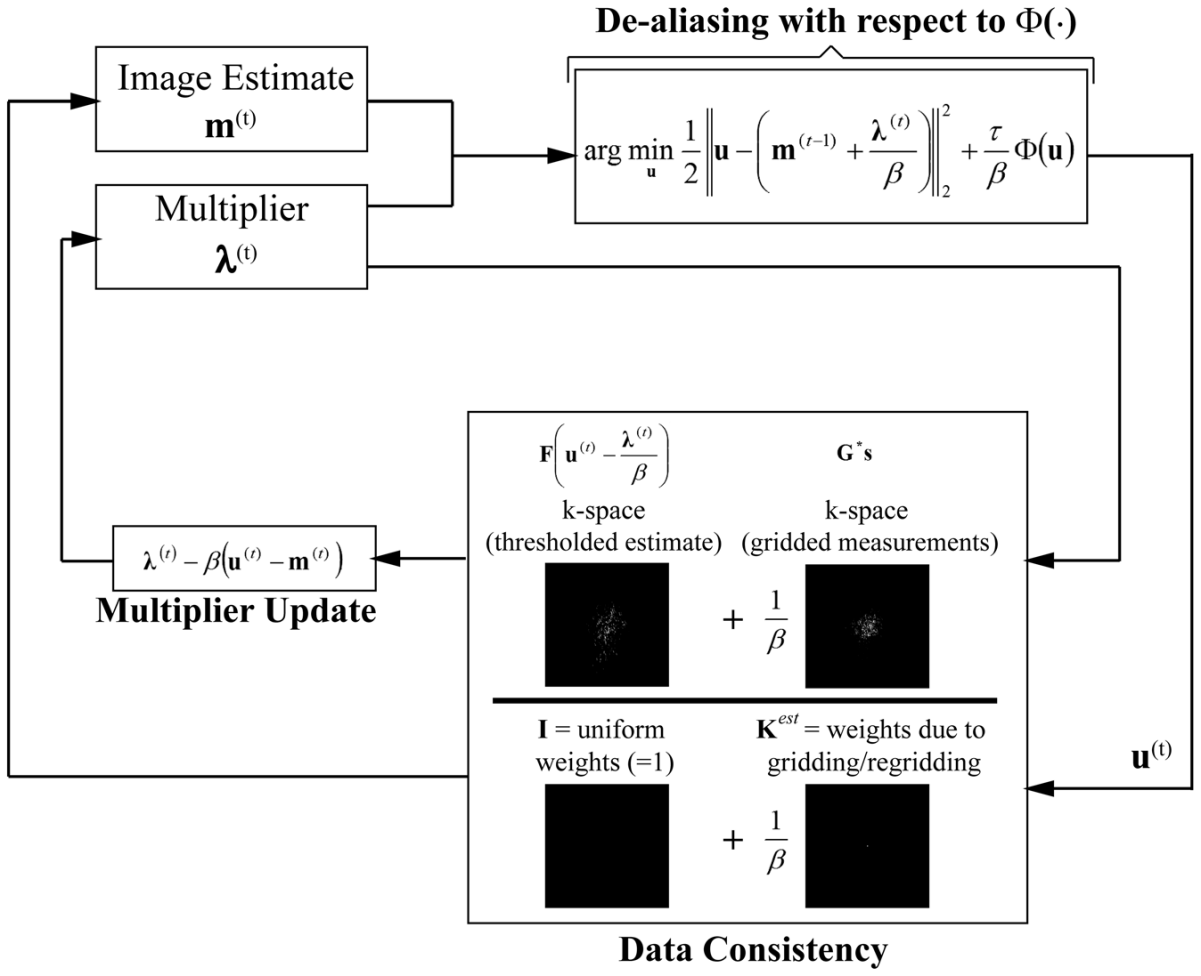
Flowchart for the proposed reconstruction algorithm for non-Cartesian acquisitions. At every iteration, the current image estimate (shifted by the multiplier) is first transformed de-aliased with respect to the sparsity constraint Φ(·) (e.g. soft-thresholding in the wavelet domain) to generate **u** ^(t)^. Then, data consistency is enforced by a weighted average of the k-space of the thresholded signal (shifted by the multiplier) and the acquired gridded k-space (G^*^s), with weights determined by *β* and **K** *_est_*, generating the new estimate **m** ^(t)^. The multiplier is also updated using **u** ^(t)^ and **m** ^(t)^. The final image is generated via de-apodization of the estimate obtained at the end of the iterative process (k-space images depict one representative slice from the volume).

The overall iterative reconstruction procedure is depicted in **Figure 1**. We note that the calculation of **K** *_est_* requires one gridding and one regridding operation. Similarly, G^*^s needs to be calculated only once prior to the iterative process, also requiring one gridding operation. Hence a total of 3 gridding and regridding operations are used in the proposed method irrespective of the number of iterations.

## Materials and Methods

All phantom and volunteer data were acquired on a 1.5-T Philips Achieva (Philips Healthcare, Best, The Netherlands) system with a 5-channel cardiac phased-array receiver coil. All in vivo studies were approved by our institutional review board and all subjects provided consent prior to participation in the study.

### Ethics Statement

The study was performed at the Beth Israel Deaconess Medical Center (BIDMC), Boston, MA, USA and was approved by the Committee on Clinical Investigations of BIDMC (Protocol No. 2013P-000231). This study was conducted with a waiver of patient consent approved by the Committee on Clinical Investigations of BIDMC.

### Reconstruction Algorithm: Implementation Details

The proposed method was implemented in MATLAB (MathWorks, Natick, MA), as well as in C++, for off-line reconstruction on a workstation with Intel (Santa Clara, CA) Core2 Quad Q9400 CPU (2.66 GHz) and 8.0 GB memory. For all reconstructions, *τ* was chosen to be 10^−7^ times the maximum (in absolute value) of **K** *_est_*. In the reconstruction, an image mask was first applied with weights inversely proportional to the de-apodization function to suppress signal from outside the region-of-interest, with *β* = 10. This served as the starting image for the iterative procedure using the Daubechies4 wavelets as the sparsifying transform. In this case, *l*_1_ soft-thresholding in wavelet-domain was used with *β* = 100. The values for *β* and *τ* were determined empirically, and were utilized in the same way for all reconstructions.

For a comparison of computational requirements, all images were also reconstructed using conventional iterative CS reconstruction method that uses gridding and regridding at every iteration with Daubechies4-wavelet-domain regularization implemented on a GPU, and in C++ [30]. The Kaiser-Bessel function with window size 4.0 was used for the convolution kernel for gridding [42]. Due to the dimensionality of the 3D radial datasets, no oversampling is used prior to gridding [30]. Both algorithms were run until a convergence criteria was met, which was defined by the relative change, 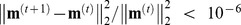. The number of iterations required to converge, as well as the time for each operation per iteration in C++ was recorded.

### Phantom Imaging

A high resolution phantom was scanned with a steady-state free precession (SSFP) sequence using a 3D radial trajectory, with 10 interleaves and 344 sample points per projection with different sampling densities of 10, 20, 30, 40 and 100%, corresponding to 289, 576, 896, 1184 and 2954 projections per interleaf respectively. The scan parameters were TR/TE/α = 3.9/1.9/60°, FOV = 240×240×240 mm^3^, and spatial resolution  = 1.4×1.4×1.4 mm^3^. The acquired 3D radial data were reconstructed using the proposed method, and the conventional iterative CS reconstruction method with gridding and regridding at every iteration. The normalized mean-squared error (MSE) with respect to the reference image with 100% sampling density, **m**_ref_, was calculated as 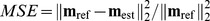, where **m**_est_ is the reconstructed image.

### In Vivo Imaging

Whole-heart MR images were acquired on 5 healthy adult subjects (32.6±16.3 years, range: 21 – 55 years, 4 women). 3D free-breathing ECG-triggered SSFP sequences were used for imaging the heart with 3D radial trajectories. A respiratory navigator with 7 mm gating window was used for gating and tracking the respiratory motion [43], where the k-space data acquired within the gating window were accepted, and the k-space data acquired outside the gating window were rejected and re-acquired until acquired within the gating window. Within the 7 mm gating window, the position of the imaging volume was adaptively adjusted using a tracking factor of 0.6. The data sets were acquired with 10 interleaves, 768 projections per interleaf and 392 sample points per projection for a sampling density of 20%. The scan parameters were as follows: TR/TE/α = 3.9/1.9/60°, FOV = 256×256×256 mm^3^, and spatial resolution  = 1.3×1.3×1.3 mm^3^. The acquired 3D radial data were reconstructed using the proposed CS method, and the conventional iterative CS reconstruction method with gridding and regridding at every iteration.

The normalized vessel sharpness and the vessel length of the right coronary artery (RCA) were measured using a Soap-Bubble tool [44] for quantitative assessment of the quality of the CS reconstruction method. Vessel sharpness scores were calculated for both sides of the vessel using Deriche algorithm [45]. Final normalized sharpness was defined as the average score of both sides divided by the center of vessel intensity. The sharpness and the length of the vessels from the two CS reconstruction techniques were compared using a paired *t*-test. A value of *P*<0.05 was considered to be statistically significant.

## Results

### Computational Requirements

**Table 1** summarizes the per iteration cost of both the conventional and the proposed CS algorithms for the reconstruction of a phantom data set with 10 interleaves, 289 projections per interleaf and 344 sample points per projection with standard C++ implementation. Due to the necessity of performing gridding and regridding at every iteration, the conventional CS algorithm has approximately 3 times the computational requirement of the proposed CS algorithm per iteration (52.48 seconds vs. 17.55 seconds). For the in-vivo datasets, the average numbers of iterations required for convergence by the different methods were 455±320 for the conventional CS technique, and 60±13 for the proposed CS technique. Thus, for in-vivo datasets, this leads to a ∼23-fold saving in the total reconstruction time on average for the proposed technique over the conventional one.

**Table 1:** Average time (in seconds) required for performing the main operations in one iteration of a C++ implementation of the CS reconstruction methods on a standard workstation with a 2.66-GHz central processing unit and 8 GB RAM, for each coil for a 3D radial data of size (*N_s_*, *N_p_*, *N_i_*) = (392, 768, 10), corresponding to 20% sampling density.

	FFT	IFFT	Gridding	Regridding	Addition (d. c.)	Matrix Inversion (d. c.)	Thresholding	Misc.	Total
**Conventional Method**	5.00	5.04	17.59	17.64	N/A	N/A	1.10	6.07	**52.48**
**Proposed Method**	5.00	5.04	N/A	N/A	0.23	0.29	1.10	5.89	**17.55**

The proposed CS technique approximately has 1/3 of the computational requirements of the conventional CS technique per iteration, due to the absence of gridding and regridding at every iteration (FFT = fast Fourier transform, IFFT = inverse fast Fourier transform, d.c. = data consistency, Misc. = miscellaneous).

### Phantom Imaging

**Figure 2:**
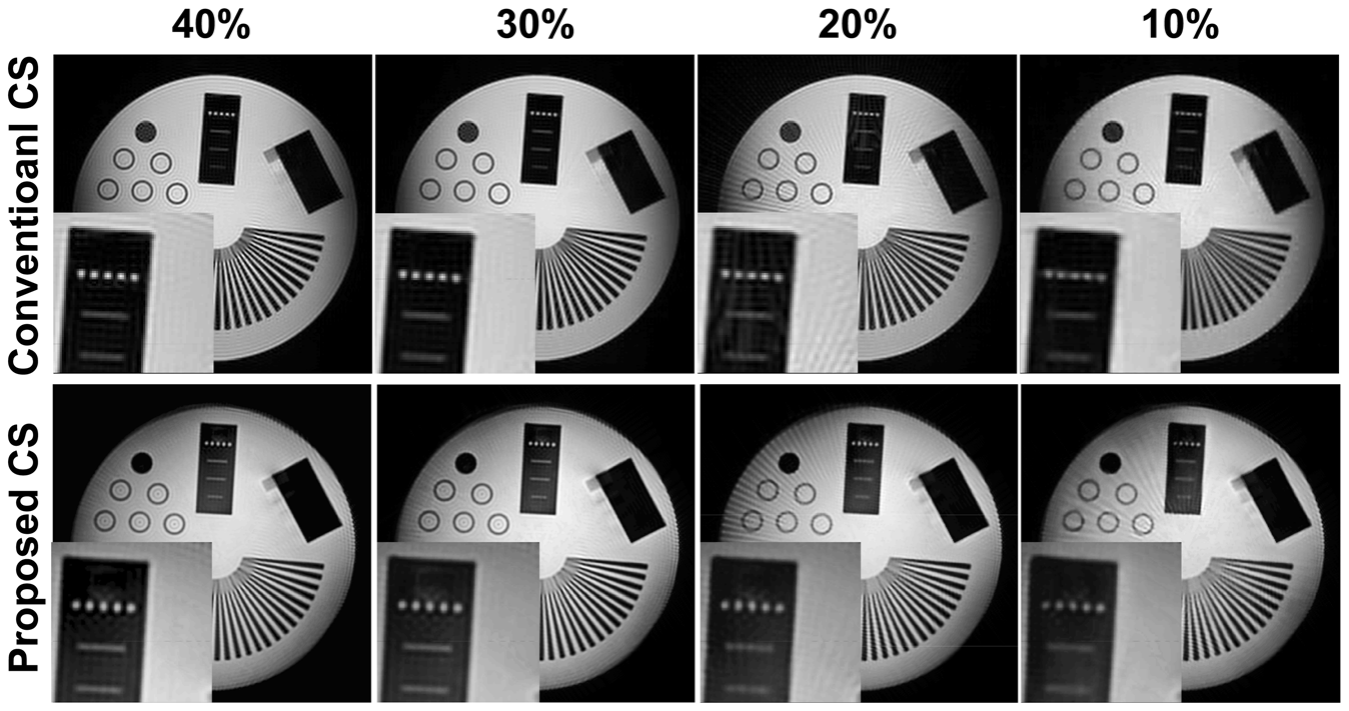
Reconstructions from 3D radial phantom imaging at 40, 30, 20 and 10% sampling densities: conventional CS reconstruction with gridding and regridding at every iteration and wavelet domain regularization (top); proposed method without gridding and regridding at every iteration and wavelet domain regularization (bottom). The details are preserved in a comparable manner between the two techniques. The proposed method exhibits more residual streaks compared to the conventional CS, apparent in the background signal in the zoomed area.

**Figure 2** depicts an example slice from the reconstruction results for the phantom imaging experiment with 40, 30, 20 and 10% sampling densities, using conventional iterative CS that utilizes gridding and regridding at every iteration, and the proposed CS method. The details are preserved in a comparable manner between the two techniques. The normalized MSE for these reconstructions were 0.006, 0.007, 0.012 and 0.017 for the conventional CS method; and 0.007, 0.008, 0.012 and 0.025 for the proposed CS method for sampling densities of 40, 30, 20 and 10% respectively. The proposed method exhibits more residual streaks compared to the conventional CS, apparent in the background signal in the zoomed area. However, the proposed technique has a clear advantage in terms of reconstruction time. The average numbers of iterations required for convergence were 45±2, 40±2, 40±1 and 40±1 for sampling densities of 10, 20, 30 and 40% respectively, indicating that the convergence behavior does not change significantly with the undersampling density.

### In Vivo Imaging

**Figure 3:**
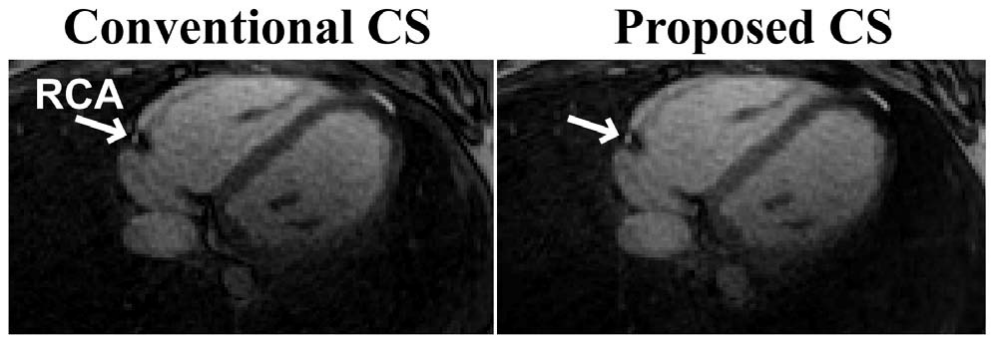
An example axial slice from a 3D radial whole heart MRI dataset at 20% sampling density, reconstructed with conventional CS reconstruction (left), and the proposed CS reconstruction (right), both with wavelet domain regularization. A cross section of the right coronary artery (RCA) is visualized clearly with both techniques.

**Figure 4:**
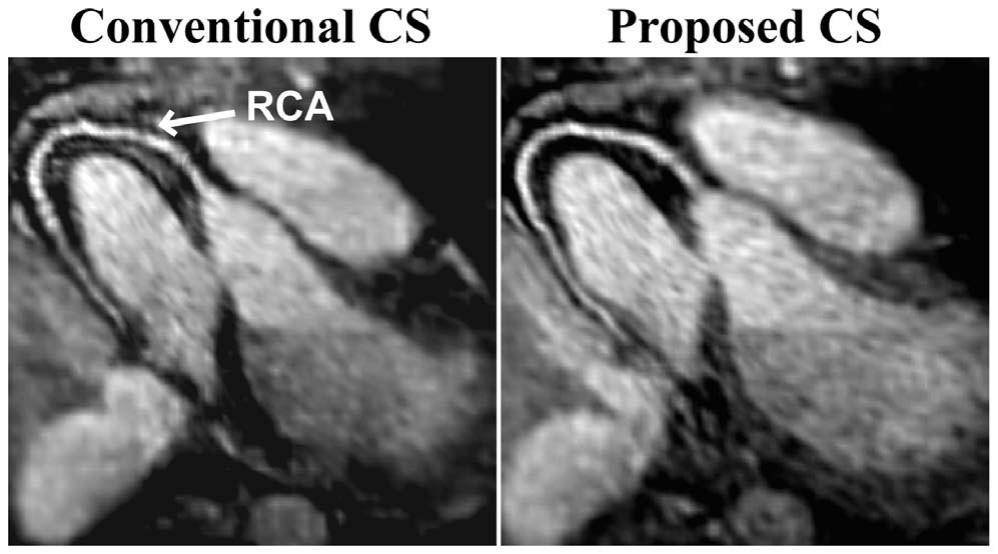
Reformatted axial images of the RCA with isotropic resolution of 1.3 mm^3^ from the whole-heart 3D radial acquisition of Figure 3 with 20% sampling density. Images are reconstructed both with the conventional CS reconstruction utilizing gridding and regridding at every iteration (left) and the proposed CS technique without gridding and regridding at every iteration (right). Both CS reconstructions employ wavelet domain regularization. Proximal, mid and distal regions of the RCA are visualized in both techniques.

**Figure 3** shows an example axial slice from a 3D whole-heart radial acquisition with 20% sampling density, reconstructed using the conventional and proposed CS techniques with wavelet-domain sparsity regularization. A cross-section of the RCA is clearly visualized with both CS techniques, which offer similar image quality and suppression of streaking artifacts that are typically associated with undersampling of radial acquisitions. We note that differences in SNR are observed in the distal RCA, likely due to residual reconstruction artifacts with the proposed method. **Figure 4** depicts reformatted axial images of the RCA from the same acquisition, reconstructed using the two CS techniques. The proximal, mid and distal portions of the RCA are visualized using both techniques even though the acquisition was with 20% sampling density. **Table 2** depicts the quantitative vessel measurements of the 3D radial whole-heart images for the five subjects. There are no significant differences between the conventional and proposed CS techniques in terms of the visualized vessel length or normalized vessel sharpness of the RCA; but the proposed technique offers a ∼23-fold saving in computational complexity.

**Table 2:** Vessel length and normalized vessel sharpness measurements for the conventional CS reconstruction with gridding and regridding at every iteration, and the proposed CS technique.

	RCA sharpness		RCA length (cm)	
Subject	Conventional CS	Proposed CS	Conventional CS	Proposed CS
1	0.555	0.554	11.62	11.70
2	0.542	0.507	6.41	6.76
3	0.481	0.496	4.07	3.70
4	0.578	0.578	10.56	10.84
5	0.323	0.278	6.22	6.22
average	0.496±0.103	0.483±0.119	7.76±3.18	7.84±3.35

There was no statistical difference between the two reconstructions in terms of vessel length (*P* = 0.30) or vessel sharpness (*P* = 0.62).

## Discussion

In this study, we have proposed an iterative CS reconstruction method for non-Cartesian trajectories, which does not require a gridding and regridding operation to be applied at every iteration. A total of three gridding/regridding operations are required, which enables the implementation of the technique with a standard MATLAB script, even for highly memory-intensive 3D radial trajectories. Phantom and in vivo cardiac MRI datasets were used to demonstrate the efficacy of the proposed technique in removing streaking artifacts, with results similar to a conventional CS implementation that has a much higher computational burden.

The main source of the computational time reduction in our proposed method is to the use of the diagonal approximation used for G^*^G. The accuracy of this approximation is important for the utility of the proposed method. One possible way to characterize the approximation accuracy is to find the closest diagonal matrix to G^*^G with respect to some distance metric (e.g. Frobenius norm). However, it is not clear how the error with respect to the specific metric propagates in the non-linear reconstruction, and what kind of artifacts and distortion it causes in the final reconstruction. Hence, we have verified our approximation by the final results of the algorithm, utilizing objective quantitative measures such as vessel sharpness and length, and the images themselves to depict the artifacts. Furthermore, since the propagation of the error is not characterized in a closed-form manner, the applicability of the technique for different configurations of trajectories warrants further study, specific to the application.

Our proposed approximation, **K** *_est_* also has an intuitive explanation: It is the result of regridding an all-ones k-space onto the spokes acquired and gridding these spokes back to a Cartesian k-space. In essence, **K** *_est_* specifies the weights associated with a particular k-space location in the gridded data G^*^s with points closer to the spokes or to the center getting a bigger weight, and it has to be calculated only once before reconstruction. Hence, the data-consistency step provides a weighted average value of the acquired gridded k-space and the k-space corresponding to the thresholded estimate (shifted by the Lagrange multiplier), normalized by the sum of weights. In contrast, for the Cartesian case, the data consistency is typically done by replacing the acquired locations in the k-space of the thresholded estimate with the acquired lines [21], [39], which is not possible in the non-Cartesian setting.

Other techniques have been proposed to approximate the gridding and regridding operations before. In the context of parallel imaging, approximations have been used both for SENSE [41] and GRAPPA [17], [46] reconstructions. These methods all rely on linear reconstructions, unlike the proposed non-linear reconstruction method. For linear methods, the effects of the approximations are easier to characterize and it is not clear whether the same conclusions extend to non-linear reconstructions. In the context of CS reconstruction for non-Cartesian MRI, other approximations have been performed [47], where the algorithm alternates between thresholding and application of the non-Cartesian GRAPPA operator. Thus data-consistency is not directly enforced, but only incorporated through the multiple-coil setup. Our method, on the other hand, enforces data-consistency using the measured values directly, corresponding to a weighted averaging scheme.

By avoiding gridding/regridding operations at every iteration, the proposed method achieves a 3-fold reduction in computational requirements, since gridding/regridding operations are the most computationally intensive part of every iteration. Furthermore, compared to an implementation of the conventional iterative CS algorithm, the proposed algorithm converges faster, in approximately 7.5-fold fewer iterations, which is due to the convergence properties of AL methods [36]. Thus, overall a ∼23-fold improvement in computational requirements is possible. All our comparisons are based on C++ implementations, where operations are performed sequentially. We note that it is possible to parallelize the gridding/regridding operations for the conventional CS technique on a GPU, as reported in [30], and implementations on different systems may lead to different reduction factors in computational requirements.

The images reconstructed with the proposed CS technique have comparable quality with those reconstructed by the conventional CS technique. Both of these techniques are effective in suppressing streaking artifacts associated with high undersampling rates for radial acquisitions. The characteristics of the artifacts for the two reconstructions are different, even though the same objective function is considered. Apart from the effects of the diagonal approximation, the changes in artifacts or reconstruction quality based on the specifics of the algorithm utilized to solve the objective function is documented both in signal processing [48], [49] and in imaging [50]. Thus, there are also variations in the quantitative measurements for the two algorithms.

The diagonal approximation in Equation [10] relies on the local nature of the gridding and regridding operators. While this is satisfied for the sampling densities considered in the kooshball acquisitions, it may not be a sufficient approximation for higher sampling densities or for smaller k-space dimensions. This was observed in our study with 100% sampled kooshball phantom datasets (data not shown). Thus, the wellness of this approximation should be validated first (e.g. by running one iteration of the algorithm) before using this algorithm for iterative reconstruction. A limitation of our paper is that we have only used this algorithm for kooshball datasets, but have not tried it for other trajectory designs, such as spiral acquisitions. Another requirement for the locality assumptions in the diagonal approximation is that the gridding kernel should have a small window size. The Kaiser-Bessel function with window of size 4 satisfies this requirement without sacrificing accuracy and without significant computational cost. However, smaller window sizes may lead to less accurate gridding, which may also cause artifacts. This was not explored in our study.

For all the images, the same reconstructions parameters were used to automate the process. Fine-tuning these values for each examination may allow further improvements in the quality of final images at the expense of a non-automated reconstruction process. We also note that wavelet domain was used for both CS reconstruction techniques. Even though we concentrated on wavelet domain reconstruction, the proposed technique allows for other regularizers such as TV regularization.

## Conclusions

We have developed an iterative reconstruction technique for non-Cartesian k-space trajectories that requires only two gridding and one regridding operations irrespective of the number of iterations, and has a fast empirical convergence rate, leading to substantial reduction in reconstruction time while providing images of similar quality compared to the conventional CS technique.
